# Structure of the archaeal chemotaxis protein CheY in a domain-swapped dimeric conformation

**DOI:** 10.1107/S2053230X19010896

**Published:** 2019-08-30

**Authors:** Karthik Shivaji Paithankar, Mathias Enderle, David C. Wirthensohn, Arthur Miller, Matthias Schlesner, Friedhelm Pfeiffer, Alexander Rittner, Martin Grininger, Dieter Oesterhelt

**Affiliations:** a Institute of Organic Chemistry and Chemical Biology, Buchmann Institute for Molecular Life Sciences, Goethe University Frankfurt, Max-von-Laue-Strasse 15, 60438 Frankfurt am Main, Germany; b Department of Membrane Biochemistry, Max Planck Institute of Biochemistry, Am Klopferspitz 18, 82152 Martinsried, Germany; c Computational Biology Group, Max Planck Institute of Biochemistry, Am Klopferspitz 18, 82152 Martinsried, Germany

**Keywords:** chemotaxis, signal transduction, response regulator, CheY, CheF, archaellum, protein evolution

## Abstract

Archaea are motile by the rotation of the archaellum. The archaellum switches between clockwise and counterclockwise rotation, and movement along a chemical gradient is possible by modulation of the switching frequency. This modulation involves the response regulator CheY and the archaellum adaptor protein CheF. In this study, two new crystal forms and protein structures of CheY are reported. In both crystal forms, CheY is arranged in a domain-swapped conformation. CheF, the protein bridging the chemotaxis signal transduction system and the motility apparatus, was recombinantly expressed, purified and subjected to X-ray data collection.

## Abstract 2

Che proteins are part of the signal transduction pathway between stimulus and response, mediated by the archaellum. The structure of the chemotaxis protein CheY was determined in two different crystal forms. The novel CheF protein that connects chemotaxis signaling to the motility apparatus was expressed and crystallized, and data were acquired by X-ray diffraction.

## 1. Introduction   

Archaea and bacteria share the ability to move in response to chemical or physical stimuli towards favorable growth conditions (Marwan & Oesterhelt, 2000 ▸; Quax, Albers *et al.*, 2018 ▸). Motility is based on the rotation of the flagellum (in bacteria) and the archaellum (in archaea; formerly known as the archaeal flagellum), respectively, and the directionality of the movement is provided by modulating the switching frequency in response to the stimulus (Armitage, 1999 ▸). The molecular basis underlying taxis is composed of two systems: chemotaxis signal transduction, which processes the external stimulus, and the flagellum/archaellum, which responds to the chemotaxis output signal.

The Che proteins, encoded by genes that cluster in genomes, constitute the chemotaxis signal transduction system. The overall mechanism of chemotaxis is conserved in archaea and bacteria (Szurmant & Ordal, 2004 ▸). Receptors, generally known as methyl-accepting chemotaxis proteins (MCPs) and referred to as halobacterial transducer proteins (Htrs) in halophilic archaea (Zhang *et al.*, 1996 ▸), sense external stimuli such as chemicals, oxygen or light. The histidine kinase CheA and the response regulator CheY form a stimulus–response coupling mechanism, generally termed the two-component system (Parkinson & Kofoid, 1992 ▸; Parkinson, 1993 ▸). CheA autophosphorylates and subsequently donates the phosphate to CheY, yielding phosphorylated CheY (CheY-P; Garrity & Ordal, 1997 ▸; Bischoff *et al.*, 1993 ▸; Rudolph & Oesterhelt, 1995 ▸; Rudolph *et al.*, 1995 ▸). The concentration of CheY-P determines the switching frequency of the flagellum or archaellum, respectively. Several Che proteins are involved in adapting (CheR, CheB, CheC, CheD and CheV; Springer & Koshland, 1977 ▸; Simms *et al.*, 1985 ▸, 1987 ▸; Stock & Koshland, 1978 ▸; Muff & Ordal, 2007 ▸; Karatan *et al.*, 2001 ▸; Schlesner *et al.*, 2012 ▸) or shutting down (CheZ, CheX, CheC and FliY; Silversmith, 2010 ▸; Sircar *et al.*, 2013 ▸) the response, the latter by removal of the phosphoryl modification from CheY-P.

In bacteria, CheY-P interacts with the flagellar motor switch protein FliM (Welch *et al.*, 1993 ▸). The CheY-P–FliM inter­action has been shown to be responsible for increasing the probability of a switch in the rotational direction of the flagellum (Berg, 2003 ▸). In archaea, no homologs of FliM have been identified, and the interaction of CheY-P with different partners in bacteria and archaea has been considered to be a factor that separates the archaeal system of motility from the bacterial system of motility.

In interactomic studies, we have recently identified three candidate proteins in *Halobacterium salinarum* that are involved in the interaction of Che and Fla proteins (Schlesner *et al.*, 2009 ▸). Analysis of deletion strains provided compelling evidence that two open reading frames in particular, OE2401F and CheF1 (OE2402F), are essential for controlling the directionality of archaellar rotation. OE2401F encodes a HEAT_PBS or HEAT family protein comprised of bihelical HEAT-like repeats. CheF1 encodes a protein from the conserved CheF-arch protein domain family (previously DUF439), which is exclusively found in Euryarchaea. *CheF* genes are consequently located in the chemotaxis gene regions and their occurrence is strictly correlated with the presence of *che* genes (Schlesner *et al.*, 2009 ▸, 2012 ▸). Protein–protein interaction analysis of the halobacterial Che proteins revealed that CheF1 directly interacts with CheY, CheD and CheC2, as well as with ArlCE (FlaCE, OE_2386R). As such, CheF1 is proposed to provide the missing function of bridging the chemotaxis signal transduction system and the motility apparatus, thereby representing the factor that connects the Che cascade, which is shared by archaea and bacteria, to the archaea-specific motility apparatus. The archaeal CheY has recently been structurally determined and in its interplay with CheF has been analyzed, providing a basal molecular bio­logical understanding of how a conserved chemotaxis system can target the entirely different motility structures in bacteria and archaea (Quax, Altegoer *et al.*, 2018 ▸). Here, we present single-crystal X-ray structures of CheY from *Pyrococcus horikoshii* (PhCheY) in two different crystal forms, and the protein purification, crystallization and X-ray data collection of CheF (PhCheF). Our data support the conservation of the bacterial and archaeal response-regulator proteins. We observe PhCheY to have a domain-swapped, pseudo-dimeric fold, which may reflect inherent properties of the protein fold but is not likely to be of physiological relevance.

## 2. Materials and methods   

### 2.1. Macromolecule production   

The coding sequences for PhCheF and PhCheY were provided by a synthetic plasmid carrying the ORFs PH0494 (PhCheF) and PH0482 (PhCheY) and were amplified by PCR with PH0494 specific primers (forward primer AAGGAGATATACATATGCCGATCTTTGAAGCCCG; reverse primer GGTGGTGGTGCTCGAGCATGCTCACCAGGCCATATTTC) and PH0482 specific primers (forward primer AAGGAGATATACATATGGCTCGTGTTCTGGTTGT; reverse primer GGTGGTGGTGCTCGAGACTAGACAGCACACGGATTCAC). In an In-Fusion cloning reaction (Clontech, Japan), the gel-purified fragments were ligated with NdeI and XhoI linearized pET-22b(+) (Novagen, USA), yielding the plasmids pDW01-1 and pDW02-1 for the expression of PhCheF and PhCheY as C-terminally His-tagged proteins. For the construction of non-His-tagged variants, we removed the His tag by inserting TAG codons upstream of the XhoI restriction site via the site-directed mutagenesis method (plasmids pDW01-2 and pDW02-2).

For heterologous expression of proteins in *Escherichia coli*, the plasmids were transformed into BL21 Gold (DE3) cells (Agilent Technologies, USA). Single colonies were used to inoculate 35 ml LB medium containing 100 µg ml^−1^ ampicillin and were incubated at 37°C for 16 h. This preculture was used to inoculate 2 l TB medium, which was grown at 37°C and 180 rev min^−1^ until the mid-to-late log phase (OD_600_ = 0.8–1.0) before inducing expression at 20°C with 0.5 m*M* IPTG. The cells were harvested after 16 h of protein expression, and the cell pellets were frozen in liquid nitrogen and stored at −80°C until further use. For purification, the cells were resuspended in appropriate buffers containing protease inhibitors (Roche, Switzerland) and DNaseI (Applichem, Germany) and were lysed using a French press. The lysates were centrifuged for 1 h at 4°C and 47 000*g*. After centrifugation, the supernatants were subjected to purification protocols. The non-His-tagged proteins PhCheF and PhCheY (in buffer H; 20 m*M* Tris–HCl pH 7.5, 100 m*M* NaCl, 5 m*M* DTT) were purified by heating the supernatant to 80°C for 20 min followed by centrifugation of the precipitated bio­molecules at 24 000*g* for 20 min. As analyzed by SDS–PAGE, this treatment precipitated most of the proteins of the *E. coli* expression host, while the thermostable proteins PhCheF and PhCheY remained soluble. Concentration of PhCheF and PhCheY and size-exclusion chromatography (SEC) using a Superdex 200 26/60 column (GE Healthcare, USA), removing the remaining proteins and soluble biomolecules (as metabolites and nucleic acids), eventually yielded proteins that were suitable for biomolecular analysis and crystallization. His-tagged proteins were purified by nickel-chelating affinity chromatography [standard protocol; wash buffer W (20 m*M* Tris–HCl pH 7.5, 100 m*M* NaCl, 20 m*M* imidazole) and buffer E (the same as buffer W but with 500 m*M* imidazole)] and SEC (buffer H) using a Superdex 200 26/60 column (GE Healthcare, USA).

For the *in vitro* pull-down assay with PhCheF and His-tagged PhCheY, 2 ml of the supernatant of each preparation (after lysis using a French press and centrifugation) were first incubated at 37°C for 15 min (in buffer W with 20 m*M* imidazole). The incubated protein mixture was then added to 2.5 ml equilibrated Ni–NTA beads (in 5–7.5 ml buffer W). After further incubation at 4°C for 1 h, the slurry was subjected to a gravity-flow column. The beads were washed five times with buffer W (one column volume per step) and the proteins were the eluted with buffer E (half a column volume per step). Fractions were collected and loaded onto a gel. The elution peak fractions were also subjected to SEC using a Superdex 200 10/300 column (GE Healthcare, USA). Ni–NTA pull-downs were also performed with 5 m*M* BeSO_4_ as well as 50 m*M* NaF in the respective buffers to obtain the BeF_3_^−^ species mimicking phosphorylated PhCheY (Lee *et al.*, 2001 ▸). Macromolecule-production information is summarized in Table 1 ▸.

### 2.2. Crystallization   

PhCheF and PhCheY were screened using commercially available screens in 96-well plates. For crystallization, pooled fractions from SEC were used. PhCheF crystallized in 0.1 *M* Tris–HCl pH 7.8, 0.1 *M* ammonium sulfate, 0.3 *M* sodium formate, 3% PGA-LM, 3% PEG 8000. His-tagged PhCheY crystallized in 0.1 *M* Tris–HCl pH 8.0, 1.2 *M* sodium malonate. Crystallization information is summarized in Table 2 ▸.

### 2.3. Data collection and processing   

Multi-wavelength anomalous dispersion (MAD) data for PhCheF were collected on beamline PX2 at the Swiss Light Source synchrotron facility, Villigen, Switzerland. The MAD data set was collected at three wavelengths: 0.9795 Å (peak), 0.9797 Å (inflection) and 0.9718 Å (remote). For this experiment, the crystal in the droplet was transferred into a cryo­solution consisting of the mother liquor supplemented with 20%(*v*/*v*) ethylene glycol for 1 min and was then cryocooled by plunging it into liquid nitrogen. X-ray diffraction data were recorded on a PILATUS 6M detector (Dectris) while the crystal was held in a gaseous N_2_ stream at 100 K.

In the case of PhCheY, data sets were collected on beamline ID14-1 at the European Synchrotron Radiation Facility, Grenoble. The crystals were also maintained at 100 K, while data were recorded on a CCD detector (ADSC Quantum Q315r). All data sets were auto-processed and merged with the *xia*2 suite (Winter *et al.*, 2013 ▸) using *XDS* (Kabsch, 2010 ▸) and *AIMLESS* (Evans & Murshudov, 2013 ▸). Data-collection and processing statistics are summarized in Table 3 ▸.

### 2.4. Structure solution and refinement   

In the case of PhCheY, two different crystal forms were obtained in the monoclinic systems *P*2 and *C*2. The solvent-content calculations for the *P*2 crystal form performed with *MATTHEWS_COEF* (Matthews, 1968 ▸) indicated a solvent content of 58% with three molecules in the asymmetric unit. In the case of the *C*2 crystal form, *MATTHEWS_COEF* indicated a solvent content of 57% with six molecules in the asymmetric unit. A search for the PhCheY sequence (UniProt accession code O58193) against the Protein Data Bank (PDB) with *BLAST* (Altschul *et al.*, 1990 ▸) revealed 71% sequence identity to *Thermotoga maritima* CheY (PDB entry 1u0s; Park *et al.*, 2004 ▸). Both PhCheY data sets were solved by the molecular-replacement method with *Phaser* (McCoy *et al.*, 2007 ▸) using a single chain of the structure with PDB code 1u0s. The resulting molecular-replacement models were refined with *REFMAC* (Murshudov *et al.*, 2011 ▸) with iterative manual model building with *Coot* (Emsley & Cowtan, 2004 ▸). Refinement statistics are summarized in Table 4 ▸. The PhCheF data could not be phased and therefore the structure could not be determined.

All structural figures were drawn using *PyMOL* (DeLano, 2002 ▸; http://www.pymol.org). Atomic coordinates and experimental structure factors for PhCheY in space groups *P*2 and *C*2 have been deposited in the PDB with accession codes 6er7 and 6exr, respectively. Raw X-ray diffraction data for both PhCheF and PhCheY are available from the Zenodo science data archive (https://doi.org/10.5281/zenodo.1148967).

## 3. Results and discussion   

### 3.1. Preparation of *P. horikoshii* CheF and CheY   

Proteins from *H. salinarum* have been expressed recombinantly in *E. coli*, but primarily as unfolded proteins that rely on the uncertainty of refolding protocols (Marg *et al.*, 2005 ▸; Grininger *et al.*, 2006 ▸). Therefore, we decided to work with CheF and CheY from *P. horikoshii* (termed PhCheF and PhCheY, respectively), which display sequence identities of 24 and 53% to CheF1 and CheY from *H. salinarum*, respectively (Supplementary Fig. S1). In addition, we expected increased thermostability of these proteins (Szilágyi & Závodszky, 2000 ▸) owing to the thermophilic lifestyle of the source (*P. horikoshii*; Kawarabayasi *et al.*, 1998 ▸).

### 3.2. Crystal structure of *P. horikoshii* CheY (PhCheY)   

Three-dimensional structures of CheY from several organisms have been determined, for example those from *E. coli* (Volz & Matsumura, 1991 ▸; Lee *et al.*, 2001 ▸), *Salmonella enterica* (Guhaniyogi *et al.*, 2006 ▸), *Thermotoga maritima* (Usher *et al.*, 1998 ▸), *Vibrio cholerae* (Biswas *et al.*, 2013 ▸) and *Helicobacter pylori* (Lam *et al.*, 2010 ▸). We aimed at determining the X-ray structure of PhCheY owing to the limited structural information on archaeal CheY [the crystal structure of a CheY-like protein from *Methanospirillum hungatei* JF-1 (PDB entry 3cg4; 31% sequence identity to PhCheY), the NMR structure of the CheY-like MTH538 from *Methanobacterium thermoautotrophicum* (PDB entry 1eiw; 31% sequence identity; Cort *et al.*, 2000 ▸) and the recent crystal structure from *Methanococcus maripaludis* (PDB entries 6ekg and 6ekh; 50% sequence identity; Quax, Altegoer *et al.*, 2018 ▸)].

**Figure 1:**
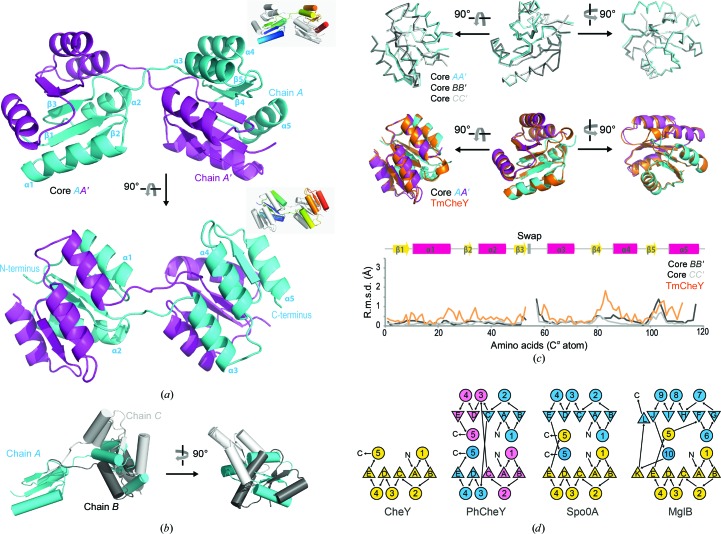
Data for the crystal form in space group *P*2 are shown. (*a*) Overall structure of PhCheY with the protomers of a dimeric structure colored cyan and magenta. PhCheY retains the overall (β/α)_5_ fold of CheY, but shows a different packing by swapping about half of the fold. Three molecules are found in the asymmetric unit. Chain *A* is shown in cyan and the symmetry-related chain *A*′ is in magenta. The chains form a total interface of 2610 Å^2^ for the *AA*′ interaction (the values are 2460 Å^2^ for the *BB*′ interaction and 2420 Å^2^ for the *CC*′ interaction). (*b*) Superposition of the polypeptide chains within the asymmetric unit. The N-terminal parts of the chains (residues 1–53) have been superimposed. Superpositions were calculated with the *LSQ* tool (least-squares fit) in *Coot* (Emsley *et al.*, 2010 ▸). (*c*) Superposition of PhCheY monomers (top; ribbon representation of backbone) and of a PhCheY pseudomonomer (reconstituted from chains *A* and *A*′) with *T. maritima* CheY (middle; PDB entry 1tmy; Usher *et al.*, 1998 ▸). R.m.s.d. plot showing deviations from a chain *A*/*A*′ PhCheY pseudomonomer (bottom). Values were calculated with the *SSM* (secondary-structure match) tool in *Coot* (Emsley *et al.*, 2010 ▸). The r.m.s.d. diagram shows the largest overall deviation in the β3–α3 loop hinge region (values exceeding 3 Å) as well as in the β5–α5 loop. (*d*) Topology diagram in the style of Fukami-Kobayashi *et al.* (1999 ▸) of the folds of CheY, the CheY homolog Spo0A and the evolutionarily related MglA.

The structures of CheY available in the PDB typically display a compact folded structural appearance. The subunits of CheY consistently show a β1/α1/β2/α2/β3/α3/β4/α4/β5/α5 globular fold of five helices flanking a five-stranded parallel β-sheet. The subdomain in the N-terminal region (referred to as the N-terminal domain; residues 1–53) has α-helices α1–α2 packing on opposite sides of β-strands β1–β3, and the C-terminal region of the molecule (referred to as the C-terminal domain; residues 61–118) has α-helices α3–α5 packing against β-strands β3–β5. In striking contrast to all of the known CheY structures, PhCheY adopts an open conformation in both crystal forms, with the two subdomains of a given subunit directed away from one another. In both of the two PhCheY structures solved in this study, two polypeptide chains assemble by pairing the N-terminal subdomain of a chain with the C-terminal subdomain of the other chain; *i.e.* the α3/β4/α4/β5/α5 part of the fold of each subunit packs with the β1/α1/β2/α2 part of the other subunit [Fig. 1 ▸(*a*)].

The crystal form with space group *P*2 contains three non­crystallographic symmetry (NCS) molecules in the asymmetric unit. Superposition of the three subunits shows differing orientations between the domains, leading to an inter-domain movement of up to 90° [Fig. 1 ▸(*b*)]. The N- and C-terminal domains of PhCheY superimpose well onto each other in the crystal structure. The closest structural homolog is CheY from *T. maritima* (Usher *et al.*, 1998 ▸; PDB entry 1tmy; 71% sequence identity, with an r.m.s.d. of around 0.7 Å and a *Z*-score of 25; Holm & Laakso, 2016 ▸). *T. maritima* CheY and PhCheY superimpose well, with the main deviation in the connecting region composed of the short helical segment of the β3–α3 loop. This region is unwound in the PhCheY structure (residues 54–60) [Fig. 1 ▸(*c*)], facilitating the inter-domain movement.

In the case of the *C*2 crystal form, there are six molecules in the asymmetric unit. Four of the subunits (*A*, *B*, *D* and *F*) interact with one another in a dimeric-type domain-swapped assembly as described above. The other two subunits, *C* and *E*, interact with their own crystallographic symmetric molecules for the dimeric assembly with swapped domains. Superposition of the N-terminal subdomain among the different subunits *A*, *B*, *C*, *D*, *E* and *F* reveals that the C-terminal domain of subunits *B*, *C*, *D*, *E* and *F* undergoes a relative movement of up to 57° with respect to the N-terminal domain. In all cases, the rotational movement of the C-terminal domain was calculated after the initial superposition of the N-terminal domain. The connecting region between the β3–α3 loop is partially disordered for the two subunits *C* and *E*. For both crystal forms an unambiguous trace of the electron density in the β3–α3 loop was verified by a feature-enhanced map (FEM; Afonine *et al.*, 2015 ▸; shown in Supplementary Fig. S2 for the *P*2 crystal form).

CheY has been discussed as the evolutionary ancestor fold of periplasmatic binding proteins, such as for example the glucose/galactose-binding protein MglA. A domain swap, producing a pseudo-dimeric fold, has been suggested to be a key event in this process, and has been observed for the CheY-like protein Spo0A (Fukami-Kobayashi *et al.*, 1999 ▸; Lewis *et al.*, 2000 ▸) [Fig. 1 ▸(*d*)].

### 3.3. Comparison of archaeal CheY from *P. horikoshii* (PhCheY) and *M. maripaludis*   

**Figure 2:**
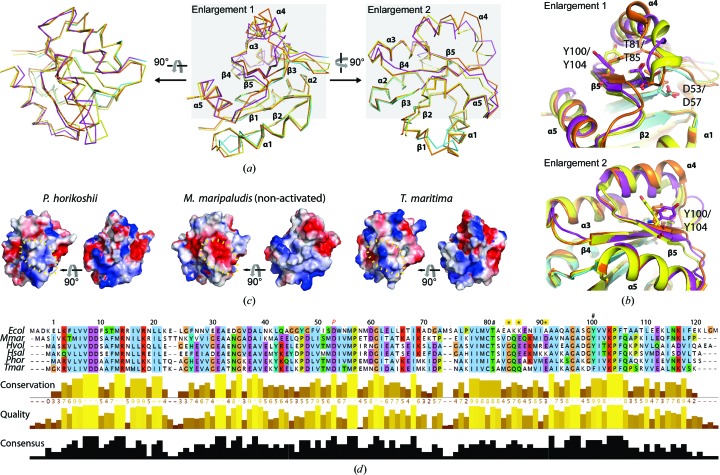
Comparative analysis of CheY. (*a*) Superposition of the PhCheY pseudomonomer (reconstituted from chains *A* and *A*′) with *M. maripaludis* CheY (PDB entries 6ekg and 6ekh; Quax, Altegoer *et al.*, 2018 ▸). Views and arrangement are as in Fig. 1 ▸(*c*). Superpositions were calculated with the *LSQ* tool (least-squares fit) in *Coot* (Emsley *et al.*, 2010 ▸). PhCheY is in magenta/cyan and BeF_3_^−^/NaF-activated and non-activated *M. maripaludis* CheY are in orange and yellow, respectively. The gray background refers to (*b*). (*b*) Enlargement of the CheY structures in the orientations indicated in (*a*). Residues Asp53/Asp57 (*P. horikoshi*/*M. maripaludis* numbering), Tyr100/Tyr104 and Thr81/Thr84 are shown in stick representation using the color code in (*a*). (*c*) Qualitative surface electrostatic representation of CheY from *P. horikoshi*, *M. maripaludis* and *T. maritima* (PDB entry 1tmy; Usher *et al.*, 1998 ▸) calculated with the vacuum electrostatics function in *PyMOL* (http://www.pymol.org) and shown in default coloring with positive potentials depicted in blue and negative potentials in red. The α4 helices including the positions of Gln85 and Glu86 (*P. horikoshi* numbering; Glu89 and Gln90 in *M. maripaludis*) are circled in yellow. (*d*) Sequence alignment in *P. horikoshi* numbering generated with *Clustal Omega* (Thompson *et al.*, 1997 ▸) and rendered in *Jalview* (Waterhouse *et al.*, 2009 ▸).

CheY from *M. maripaludis* has recently been structurally analyzed in the BeF_3_^−^/NaF activated state, mimicking phosphorylation of the active aspartate Asp57 (Asp53 in PhCheY), and in the non-activated state (Quax, Altegoer *et al.*, 2018 ▸). The structural comparison revealed a repositioning of helix α4 accompanied by the displacement of the α4–β4 loop upon BeF_3_^−^ binding, overall corresponding well to the structural changes that occur in *E. coli* and *T. maritima* CheY during activation (Lee *et al.*, 2001 ▸; Ahn *et al.*, 2013 ▸). The ‘back-swapped’ PhCheY pseudomonomer (reconstituted from chains *A* and *A*′) structure compares well with the structure of the non-activated CheY from *M. maripaludis* (r.m.s.d. of 1.1 Å, *Z*-score 22.1; Holm & Laakso, 2016 ▸) [Fig. 2 ▸(*a*)]. The positions of the active Asp53, as well as Thr81 and Tyr100, involved in translating the phosphorylation signal to a physiological output (Tyr–Thr coupling; Zhu *et al.*, 1996 ▸), also superimpose well, although Tyr100 adopts a different rotamer position [Fig. 2 ▸(*b*)]. The accumulated negative charge at the N-terminal region of helix α4 in *M. maripaludis* CheY has been suggested to provide an archaea-specific interface for CheF interaction (Quax, Altegoer *et al.*, 2018 ▸). As indicated by a qualitative surface-potential representation [Fig. 2 ▸(*c*)], PhCheY may be less negatively charged than *M. maripaludis* CheF, since it lacks a negatively charged amino acid at the position equivalent to Asp88 (Gly84 in PhCheY), as is also the case for other archaeal CheYs [Fig. 2 ▸(*d*)]. Glu91 is in a different rotamer position in PhCheY and may also contribute to the less developed negative surface potential [see Fig. 2 ▸(*c*)].

### 3.4. Biochemical characterization of interactions of CheF and CheY   

**Figure 3:**
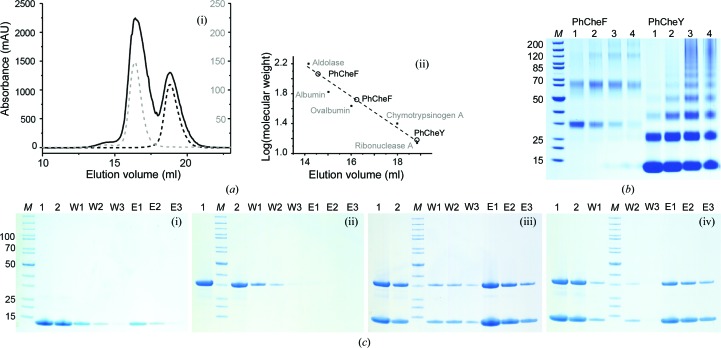
Interaction studies using size-exclusion chromatography (SEC) and Ni–NTA pull-down. (*a*) (i) SEC profiles of PhCheF (39.7 kDa; gray dashed line), PhCheY (13.1 kDa; black dashed line) and a 1:1 stoichiometric mixture of proteins (black line). (ii) Calibration with ribonuclease (13.7 kDa), chymotrypsinogen A (25 kDa), ovalbumin (43 kDa), albumin (66.2 kDa) and aldolase (158 kDa). The peaks for PhCheY and PhCheF correspond to apparent molecular weights of 15 kDa and 41 and 127 kDa, respectively (calculated molecular weights of 13.1 and 39.7 kDa, respectively). The higher oligomeric species seen for PhCheF corresponds in its apparent molecular weight to a dimeric or trimeric complex. A mixture of PhCheF and PhCheY does not form complexes under the conditions of the experiment. (*b*) Coomassie-stained SDS–PAGE gel (NuPAGE, 4–12%, Invitrogen, USA) of cross-linking studies with glutaraldehyde. Samples 1–4 reflect different incubation times before quenching (1, 2, 5 and 10 min). For PhCheF, a pronounced dimeric species appears under moderate cross-linking conditions, while PhCheY seems to successively polymerize from monomers. The SDS–PAGE image was modified in contrast. (*c*) Coomassie-stained SDS–PAGE gel (NuPAGE, 4–12%, Invitrogen, USA) of the Ni–NTA pull-down assay with PhCheF and His-tagged PhCheY. Gels (i) and (ii) show controls with PhCheY and PhCheF, respectively; gels (iii) and (iv) show pull-down with nontreated (iii) and BeF_3_^−^-treated (iv) PhCheY. PhCheF elutes with PhCheY in (iii) and (iv), suggesting specific interaction. Lane *M*, marker (PageRuler, unstained protein ladder; Thermo Scientific, USA); lane 1, control (samples incubated without Ni beads); lane 2, control (supernatant after incubation with Ni beads); lanes W1, W2 and W3, wash fractions; lanes E1, E2 and E3, elution fractions. The SDS–PAGE images were modified in contrast.

A difficult aspect in interaction studies of CheF and CheY is that CheY is supposed to interact with CheF in a phosphorylated state (CheY-P), which is however not accessible from recombinant expression. As a generally accepted treatment for mimicking phosphorylated states, we therefore incubated PhCheY with 5 m*M* BeSO_4_ and 30 m*M* NaF to modify PhCheY with the phosphoryl analog BeF_3_^−^ (Lee *et al.*, 2001 ▸). The elution profiles did not reveal higher apparent molecular masses when CheY and CheF were eluted together, indicating no complex formation [Fig. 3 ▸(*a*)]. While cross-linking with glutaraldehyde also failed in tracing interaction of the proteins under the selected conditions [Fig. 3 ▸(*b*)], an Ni–NTA pull-down assay eventually allowed the specific interaction of the proteins to be monitored by His-tagged PhCheY retaining PhCheF [Fig. 3 ▸(*c*)] during chromatographic elution. Both conditions, BeF_3_^−^-free and BeF_3_^−^-treated PhCheY, gave similar elution profiles. The complex observed in Ni–NTA pull-downs was not preserved during SEC. From Coomassie-stained SDS–PAGE in Ni–NTA pull-downs, PhCheY appears to be present in a molar excess, which might however result from nonsaturated PhCheY under the experimental conditions. We note that SEC showed the proteins to be monomeric after purification, with PhCheF having some tendency for the formation of a higher oligomeric species under the chosen conditions [see Fig. 3 ▸(*a*)]. Data collected during the biochemical characterization of CheY and CheF therefore do not support a physiological relevance of dimeric CheY species, as are observed in crystal structures.

## 4. Discussion   

Two-component systems regulate a variety of fundamental processes in metabolism and motility, as well as a set of more specialized processes such as in virulence and development (Zschiedrich *et al.*, 2016 ▸). A prototypical response regulator is composed of two domains: a receiver domain and an output domain. The receiver domain operates in a highly conserved mode of accepting a phosphoryl modification from the histidine kinase and forwarding this information to an effector domain. The effector domain triggers the output response, and the variety of effector domains allows a large number of responses regulated by two-component signals (Gao & Stock, 2009 ▸).

Owing to its important role in signal transduction, CheY has been intensively studied in recent decades. CheY proteins have been characterized as standalone proteins, resting in an equilibrium of non-activated and activated states that is shifted in response to phosphorylation (Lowry *et al.*, 1994 ▸; Lee *et al.*, 2001 ▸; Gardino & Kern, 2007 ▸). CheY is consistently described as monomeric even at high concentrations and to be independent of the phosphorylation state. As the phosphorylated state is inherently unstable, with half-lives from seconds to several hours under ambient conditions (Swanson *et al.*, 1996 ▸; Sanna *et al.*, 1995 ▸), analysis of the conformational state of activated CheY is complicated, however, and for structural studies phosphorylation was mainly just mimicked by using BeF_3_^−^-containing buffers (Lee *et al.*, 2001 ▸). In the current idea of a working mode for CheY, the activated state is read out by a subtly changed protein surface (Lee *et al.*, 2001 ▸; Gao & Stock, 2009 ▸; Quax, Altegoer *et al.*, 2018 ▸).

While CheY has been broadly characterized in structure and function, a subdomain swap, as observed in PhCheY, has not been reported before. However, it was found that the CheY α/β protein fold itself does allow domain swapping. Dimerization by domain swapping has been observed for the CheY-homologous sporulation response regulator Spo0A, although the physiological relevance of this structure was questioned in this case owing to nonphysiological crystallization at low pH (Lewis *et al.*, 2000 ▸). Further, the pseudo-dimeric fold of periplasmatic binding proteins such as MglB [see Fig. 1 ▸(*d*)] has been suggested to have evolved from a CheY-like ancestor protein by a domain swap (Fukami-Kobayashi *et al.*, 1999 ▸). When considering structural and conformational properties of the CheY fold, it is also informative to regard the folding properties of CheY. Studies on CheY revealed a heterogeneous folding trajectory, which is composed of N- and C-terminal subdomain folding in a hierarchical fashion with subdomain borders found swapped as in this study (Hills & Brooks, 2008 ▸, López-Hernández & Serrano, 1996 ▸). Both the structural properties of the CheY fold and the folding kinetics of CheY manifest the view of CheY being composed of two separate subdomains.

The assessment of the physiological relevance of the domain-swapped dimeric PhCheY is strongly connected to the question of whether reversible domain swapping could occur on a time scale that is relevant for signaling. To switch between a monomeric and a dimeric state, an interface of roughly 1300 Å^2^ between the subdomains of PhCheY would need to dissociate and reassociate in response to phosphorylation. Considering that the active aspartate (Asp53) is located in a positionally variable loop, it is unlikely that phosphorylation could efficiently induce the dissociation of the fold and enrich a ‘swapped’ form. Therefore, a physiological role for a domain-swapped dimeric PhCheY has to be ruled out. A physiological role of a domain-swapped dimer would also contradict the current understanding of the function of CheY (Quax, Altegoer *et al.*, 2018 ▸).

Crystallization of PhCheY as a domain-swapped dimer can rather be explained as follows: PhCheY is monomeric in solution, as suggested by SEC [see Fig. 3 ▸(*a*)], and in SDS–PAGE of cross-linking experiments [see Fig. 3 ▸(*b*)]. Owing to a putative cold-induced destabilization of PhCheY (*P. horikoshii* grows at 98°C), and putatively further induced by the crystallization conditions, the PhCheY fold disassembles into an open subdomain-dissociated conformation. At the high protein concentration in the crystallization drop, PhCheY can then reassemble from this open state with a partner polypeptide to form a domain-swapped dimeric assembly that is eventually removed from the monomer–dimer equilibrium by crystallization.

It is surely exciting to further reveal the different structural appearances of archaeal and bacterial CheY in order to disclose the principles of diversification in bacterial and archaeal chemotaxis/motility systems, which is then further pronounced by CheY interacting with FliM in bacteria and with CheF in archaea (Szurmant & Ordal, 2004 ▸; Albers & Jarrell, 2015 ▸; Schlesner *et al.*, 2009 ▸; Quax, Albers *et al.*, 2018 ▸).

## 5. Related literature   

The following references are cited in the supporting information to this article: Albers & Jarrell (2018 ▸), Banerjee *et al.* (2013 ▸, 2015 ▸), Briegel *et al.* (2017 ▸), Buchan *et al.* (2013 ▸), Chaudhury *et al.* (2016 ▸, 2018 ▸), Cohen-Krausz & Trachtenbergm (2002 ▸), Daum *et al.* (2017 ▸), Faguy *et al.* (1994 ▸), Frishman & Argos (1995 ▸), Gerl & Sumper (1988 ▸), Ghosh *et al.* (2011 ▸), Jarrell & McBride (2008 ▸), Kinosita *et al.* (2016 ▸), Kupper *et al.* (1994 ▸), Meshcheryakov & Wolf (2016 ▸), Patenge *et al.* (2001 ▸), Peabody *et al.* (2003 ▸), Reindl *et al.* (2013 ▸), Speranskii *et al.* (1996 ▸), Streif *et al.* (2008 ▸) and Thomas & Jarrell (2001 ▸).

## Supplementary Material

PDB reference: PhCheY, 6er7

PDB reference: 6exr

Supplementary Figures. DOI: 10.1107/S2053230X19010896/no5164sup1.pdf

Raw diffraction data for PhCheF and PhCheY.: https://doi.org/10.5281/zenodo.1148967

## Figures and Tables

**Table 1:** Macromolecule-production information Sequences of PhCheF and PhCheY are available from UniProt with accession codes O58230 and O58193, respectively.

	PhCheF	PhCheY
Source organism	*P. horikoshii*	*P. horikoshii*
DNA source	Genomic DNA	Genomic DNA
Forward primer	AAGGAGATATACATATGCCGATCTTTGAAGCCCG	AAGGAGATATACATATGGCTCGTGTTCTGGTTGT
Reverse primer	GGTGGTGGTGCTCGAGCATGCTCACCAGGCCATATTTC	GGTGGTGGTGCTCGAGACTAGACAGCACACGATTCAC
Expression vector	pET-22b(+)	pET-22b(+)
Expression host	*E. coli*	*E. coli*
Complete amino-acid sequence of the construct produced	MPIFEARVKVGISSSWVTSRKVSWRDAIAQIESDRIVVKYLKMGEVVGEDSFPFSALIDLGVRIPDELKLNPEKDHFGIKFYIPGRGELLVIFTIEENLLIYDEKKFSEFVHKVFEVLINGKTVMLQLARIIGGAVNMESKWEEGWLRVIKVKSARTQKTERSIVVIIKDKRPVSIFSDLEDIEIEEVDMNGKRVRAWKIRHFHIDQSVTSYLYIPDKQTQLYVLRYLLKYNPAIMEFIMKVSDDFPTLKSEFQEIMEKEIKELEALDEMEKQILVALYSGINPLELHQFLGVSEKEIEEIYDRMIDKGLLKIVMIRKIVDLTNEGRKIVNKLLKYGLVSM	MARVLVVDDAAFMRMLLKKILTQAGHEVVGEASNGKEAVEKYKQLKPDLVTMDIVMPEMDGITAVKEIMKIDPNAKIIMITAVGQEAKVMEALKSGAKGYIVKPFQAQKVIEEVNRVLSS

**Table 2:** Crystallization

	PhCheF	PhCheY
Method	Vapor diffusion, hanging drop	Vapor diffusion, hanging drop
Plate type	24-well plate	24-well plate
Temperature (K)	295	295
Protein concentration (mg ml^−1^)	10	10
Buffer composition of protein solution	20 m*M* Tris–HCl pH 7.5, 100 m*M* NaCl, 5 m*M* DTT	20 m*M* Tris–HCl pH 7.5, 100 m*M* NaCl
Composition of reservoir solution	0.1 *M* Tris–HCl pH 7.8, 0.1 *M* ammonium sulfate, 0.3 *M* sodium formate, 3% PGA-LM, 3% PEG 8000	0.1 *M* Tris–HCl pH 8.0, 1.2 *M* sodium malonate
Volume and ratio of drop	1:1	1:1
Volume of reservoir (µl)	750	750

**Table 3:** Data collection and processing Values in parentheses are for the outer shell. All data were processed to a CC_1/2_ of 0.5.

	PhCheF, peak	PhCheF, inflection	PhCheF, remote	PhCheY	PhCheY
Diffraction source	SLS	SLS	SLS	ESRF	ESRF
Wavelength (Å)	0.9795	0.9797	0.9718	0.9334	0.9334
Temperature (K)	100	100	100	100	100
Detector	PILATUS 6M	PILATUS 6M	PILATUS 6M	ADSC Quantum Q315r	ADSC Quantum Q315r
Rotation range per image (°)	0.25	0.25	0.25	1	1
Total rotation range (°)	360	360	360	360	360
Exposure time per image (s)	0.25	0.25	0.25	5	5
Space group	*P*2_1_	*P*2_1_	*P*2_1_	*P*2	*C*2
*a*, *b*, *c* (Å)	50.7, 188.7, 58.3	50.7, 188.7, 58.3	50.7, 188.7, 58.3	53.2, 65.7, 72.9	109.14, 124.38, 73.42
α, β, γ (°)	90, 113, 90	90, 113, 90	90, 113, 90	90, 111, 90	90, 112, 90
Resolution range (Å)	68.2–2.9 (3.0–2.9)	68.2–2.9 (3.0–2.9)	68.2–2.9 (3.0–2.9)	34–2.6 (2.7–2.6)	50–2.16 (2.20–2.16)
Total No. of reflections	278224	140306	151483	107515	355436
No. of unique reflections	21344	20363	21847	14172	11154
Completeness (%)	100 (100)	100 (100)	100 (100)	100 (99)	100 (99)
Multiplicity	7 (7)	7 (7)	7 (7)	7 (7)	7 (5)
〈*I*/σ(*I*)〉	28 (3.6)	22 (2)	23 (2.5)	38 (3)	22 (1.2)†
*R*_p.i.m._	0.02 (0.8)	0.03 (0.3)	0.03 (0.5)	0.01 (0.4)	0.02 (0.6)
*R*_meas_	0.06 (0.8)	0.06 (0.9)	0.06 (0.9)	0.03 (0.8)	0.02 (1.6)
Overall *B* factor from Wilson plot (Å^2^)	86	91	88	68	41

† 〈*I*/σ(*I*)〉 is 2.0 at a resolution of 2.24 Å.

**Table 4:** Structure refinement for CheY Values in parentheses are for the outer shell.

	*P*2	*C*2
Resolution range (Å)	34–2.6 (2.70–2.60)	50–2.2 (2.20–2.16)
*R*_cryst_/*R*_free_	0.21/0.26	0.24/0.27
No. of non-H atoms	2688	11114
Protein residues in the asymmetric unit	351	698
R.m.s. deviations		
Bonds (Å)	0.01	0.01
Angles (°)	1.6	1.8
Average *B* factors (Å^2^)		
Protein	92	54
Ramachandran plot		
Favored regions (%)	98.5	99.12
Allowed regions (%)	1.5	0.88
Outliers (%)	0	0

## References

[bb99] Afonine, P. V., Moriarty, N. W., Mustyakimov, M., Sobolev, O. V., Terwilliger, T. C., Turk, D., Urzhumtsev, A. & Adams, P. D. (2015). *Acta Cryst.* D**71**, 646–666. 10.1107/S1399004714028132 PMC4356370 25760612

[bb1] Ahn, D.-R., Song, H., Kim, J., Lee, S. & Park, S. (2013). *Int. J. Biol. Macromol.* **54**, 76–83. 10.1016/j.ijbiomac.2012.12.003 23237794

[bb2] Albers, S. V. & Jarrell, K. F. (2015). *Front. Microbiol.* **6**, 23. 10.3389/fmicb.2015.00023 PMC4307647 25699024

[bb3] Albers, S. V. & Jarrell, K. F. (2018). *Trends Microbiol.* **26**, 351–362. 10.1016/j.tim.2018.01.004 29452953

[bb4] Altschul, S. F., Gish, W., Miller, W., Myers, E. W. & Lipman, D. J. (1990). *J. Mol. Biol.* **215**, 403–410. 10.1016/S0022-2836(05)80360-2 2231712

[bb5] Armitage, J. P. (1999). *Adv. Microb. Physiol.* **41**, 229–289. 10.1016/s0065-2911(08)60168-x 10500847

[bb6] Banerjee, A., Neiner, T., Tripp, P. & Albers, S. V. (2013). *FEBS J.* **280**, 6141–6149. 10.1111/febs.12534 24103130

[bb7] Banerjee, A., Tsai, C.-L., Chaudhury, P., Tripp, P., Arvai, A. S., Ishida, J. P., Tainer, J. A. & Albers, S.-V. (2015). *Structure*, **23**, 863–872. 10.1016/j.str.2015.03.001 PMC4425475 25865246

[bb8] Berg, H. C. (2003). *Annu. Rev. Biochem.* **72**, 19–54. 10.1146/annurev.biochem.72.121801.161737 12500982

[bb9] Bischoff, D. S., Bourret, R. B., Kirsch, M. L. & Ordal, G. W. (1993). *Biochemistry*, **32**, 9256–9261. 10.1021/bi00086a035 8369293

[bb10] Biswas, M., Dey, S., Khamrui, S., Sen, U. & Dasgupta, J. (2013). *PLoS One*, **8**, e73923. 10.1371/journal.pone.0073923 PMC3774744 24066084

[bb11] Briegel, A., Oikonomou, C. M., Chang, Y.-W., Kjaer, A., Huang, A. N., Kim, K. W., Ghosal, D., Nguyen, H. H., Kenny, D., Ogorzalek Loo, R. R., Gunsalus, R. P. & Jensen, G. J. (2017). *EMBO Rep.* **18**, 1660–1670. 10.15252/embr.201744070 PMC5579351 28729461

[bb12] Buchan, D. W. A., Minneci, F., Nugent, T. C. O., Bryson, K. & Jones, D. T. (2013). *Nucleic Acids Res.* **41**, W349–W357. 10.1093/nar/gkt381 PMC3692098 23748958

[bb13] Chaudhury, P., Neiner, T., D’Imprima, E., Banerjee, A., Reindl, S., Ghosh, A., Arvai, A. S., Mills, D. J., van der Does, C., Tainer, J. A., Vonck, J. & Albers, S.-V. (2016). *Mol. Microbiol.* **99**, 674–685. 10.1111/mmi.13260 PMC5019145 26508112

[bb14] Chaudhury, P., van der Does, C. & Albers, S.-V. (2018). *PeerJ*, **6**, e4984. 10.7717/peerj.4984 PMC6011876 29938130

[bb15] Cohen-Krausz, S. & Trachtenberg, S. (2002). *J. Mol. Biol.* **321**, 383–395. 10.1016/s0022-2836(02)00616-2 12162953

[bb16] Cort, J. R., Yee, A., Edwards, A. M., Arrowsmith, C. H. & Kennedy, M. A. (2000). *J. Mol. Biol.* **302**, 189–203. 10.1006/jmbi.2000.4052 10964569

[bb17] Daum, B., Vonck, J., Bellack, A., Chaudhury, P., Reichelt, R., Albers, S.-V., Rachel, R. & Kuhlbrandt, W. (2017). *Elife*, **6**, e27470. 10.7554/eLife.27470 PMC5517150 28653905

[bb18] DeLano, W. L. (2002). *CCP4 Newsl. Protein Crystallogr.* **40**, 11.

[bb20] Emsley, P. & Cowtan, K. (2004). *Acta Cryst.* D**60**, 2126–2132. 10.1107/S0907444904019158 15572765

[bb21] Emsley, P., Lohkamp, B., Scott, W. G. & Cowtan, K. (2010). *Acta Cryst.* D**66**, 486–501. 10.1107/S0907444910007493 PMC2852313 20383002

[bb22] Evans, P. R. & Murshudov, G. N. (2013). *Acta Cryst.* D**69**, 1204–1214. 10.1107/S0907444913000061 PMC3689523 23793146

[bb23] Faguy, D. M., Jarrell, K. F., Kuzio, J. & Kalmokoff, M. L. (1994). *Can. J. Microbiol.* **40**, 67–71. 10.1139/m94-011 7908603

[bb97] Frishman, D. & Argos, P. (1995). *Proteins*, **23**, 566–579. 10.1002/prot.340230412 8749853

[bb24] Fukami-Kobayashi, K., Tateno, Y. & Nishikawa, K. (1999). *J. Mol. Biol.* **286**, 279–290. 10.1006/jmbi.1998.2454 9931266

[bb25] Gao, R. & Stock, A. M. (2009). *Annu. Rev. Microbiol.* **63**, 133–154. 10.1146/annurev.micro.091208.073214 PMC3645274 19575571

[bb26] Gardino, A. K. & Kern, D. (2007). *Methods Enzymol.* **423**, 149–165. 10.1016/S0076-6879(07)23006-X 17609130

[bb27] Garrity, L. F. & Ordal, G. W. (1997). *Microbiology*, **143**, 2945–2951. 10.1099/00221287-143-9-2945 12094812

[bb28] Gerl, L. & Sumper, M. (1988). *J. Biol. Chem.* **263**, 13246–13251. 3417656

[bb29] Ghosh, A., Hartung, S., van der Does, C., Tainer, J. A. & Albers, S.-V. (2011). *Biochem. J.* **437**, 43–52. 10.1042/BJ20110410 PMC3213642 21506936

[bb30] Grininger, M., Zeth, K. & Oesterhelt, D. (2006). *J. Mol. Biol.* **357**, 842–857. 10.1016/j.jmb.2005.12.072 16460756

[bb31] Guhaniyogi, J., Robinson, V. L. & Stock, A. M. (2006). *J. Mol. Biol.* **359**, 624–645. 10.1016/j.jmb.2006.03.050 PMC3666561 16674976

[bb32] Hills, R. D. Jr & Brooks, C. L. (2008). *J. Mol. Biol.* **382**, 485–495. 10.1016/j.jmb.2008.07.007 PMC2564871 18644380

[bb33] Holm, L. & Laakso, L. M. (2016). *Nucleic Acids Res.* **44**, W351–W355. 10.1093/nar/gkw357 PMC4987910 27131377

[bb34] Jarrell, K. F. & McBride, M. J. (2008). *Nature Rev. Microbiol.* **6**, 466–476. 10.1038/nrmicro1900 18461074

[bb35] Kabsch, W. (2010). *Acta Cryst.* D**66**, 125–132. 10.1107/S0907444909047337 PMC2815665 20124692

[bb36] Karatan, E., Saulmon, M. M., Bunn, M. W. & Ordal, G. W. (2001). *J. Biol. Chem.* **276**, 43618–43626. 10.1074/jbc.M104955200 11553614

[bb37] Kawarabayasi, Y., Sawada, M., Horikawa, H., Haikawa, Y., Hino, Y., Yamamoto, S., Sekine, M., Baba, S.-I., Kosugi, H., Hosoyama, A., Nagai, Y., Sakai, M., Ogura, K., Otsuka, R., Nakazawa, H., Takamiya, M., Ohfuku, Y., Funahashi, T., Tanaka, T., Kudoh, Y., Yamazaki, J., Kushida, N., Oguchi, A., Aoki, K.-I. & Kikuchi, H. (1998). *DNA Res.* **5**, 55–76. 10.1093/dnares/5.2.55 9679194

[bb38] Kinosita, Y., Uchida, N., Nakane, D. & Nishizaka, T. (2016). *Nature Microbiol.* **1**, 16148. 10.1038/nmicrobiol.2016.148 27564999

[bb39] Kupper, J., Marwan, W., Typke, D., Grünberg, H., Uwer, U., Gluch, M. & Oesterhelt, D. (1994). *J. Bacteriol.* **176**, 5184–5187. 10.1128/jb.176.16.5184-5187.1994 PMC196367 8051038

[bb40] Lam, K. H., Ling, T. K. W. & Au, S. W. (2010). *J. Bacteriol.* **192**, 2324–2334. 10.1128/JB.00603-09 PMC2863492 20207758

[bb41] Lee, S.-Y., Cho, H. S., Pelton, J. G., Yan, D., Berry, E. A. & Wemmer, D. E. (2001). *J. Biol. Chem.* **276**, 16425–16431. 10.1074/jbc.M101002200 11279165

[bb42] Lewis, R. J., Muchová, K., Brannigan, J. A., Barák, I., Leonard, G. & Wilkinson, A. J. (2000). *J. Mol. Biol.* **297**, 757–770. 10.1006/jmbi.2000.3598 10731426

[bb43] López-Hernández, E. & Serrano, L. (1996). *Fold. Des.* **1**, 43–55. 9079363

[bb44] Lowry, D. F., Roth, A. F., Rupert, P. B., Dahlquist, F. W., Moy, F. J., Domaille, P. J. & Matsumura, P. (1994). *J. Biol. Chem.* **269**, 26358–26362. 7929354

[bb45] Marg, B. L., Schweimer, K., Sticht, H. & Oesterhelt, D. (2005). *Biochemistry*, **44**, 29–39. 10.1021/bi0485169 15628843

[bb46] Marwan, W. & Oesterhelt, D. (2000). *ASM News*, **66**, 83–89.

[bb47] Matthews, B. W. (1968). *J. Mol. Biol.* **33**, 491–497. 10.1016/0022-2836(68)90205-2 5700707

[bb48] McCoy, A. J., Grosse-Kunstleve, R. W., Adams, P. D., Winn, M. D., Storoni, L. C. & Read, R. J. (2007). *J. Appl. Cryst.* **40**, 658–674. 10.1107/S0021889807021206 PMC2483472 19461840

[bb49] Meshcheryakov, V. A. & Wolf, M. (2016). *Protein Sci.* **25**, 1147–1155. 10.1002/pro.2932 PMC4941775 27060465

[bb50] Muff, T. J. & Ordal, G. W. (2007). *J. Biol. Chem.* **282**, 34120–34128. 10.1074/jbc.M706432200 17908686

[bb51] Murshudov, G. N., Skubák, P., Lebedev, A. A., Pannu, N. S., Steiner, R. A., Nicholls, R. A., Winn, M. D., Long, F. & Vagin, A. A. (2011). *Acta Cryst.* D**67**, 355–367. 10.1107/S0907444911001314 PMC3069751 21460454

[bb98] Park, S.-Y., Beel, B. D., Simon, M. I., Bilwes, A. M. & Crane, B. R. (2004). *Proc. Natl Acad. Sci. USA*, **101**, 11646–11651. 10.1073/pnas.0401038101 PMC511033 15289606

[bb52] Parkinson, J. S. (1993). *Cell*, **73**, 857–871. 10.1016/0092-8674(93)90267-t 8098993

[bb53] Parkinson, J. S. & Kofoid, E. C. (1992). *Annu. Rev. Genet.* **26**, 71–112. 10.1146/annurev.ge.26.120192.000443 1482126

[bb54] Patenge, N., Berendes, A., Engelhardt, H., Schuster, S. C. & Oesterhelt, D. (2001). *Mol. Microbiol.* **41**, 653–663. 10.1046/j.1365-2958.2001.02542.x 11532133

[bb55] Peabody, C. R., Chung, Y. J., Yen, M.-R., Vidal-Ingigliardi, D., Pugsley, A. P. & Saier, M. H. Jr (2003). *Microbiology*, **149**, 3051–3072. 10.1099/mic.0.26364-0 14600218

[bb56] Quax, T. E. F., Albers, S.-V. & Pfeiffer, F. (2018). *Emerg. Top. Life Sci.* **2**, 535–546. 10.1042/ETLS20180089 PMC7289035 33525831

[bb57] Quax, T. E. F., Altegoer, F., Rossi, F., Li, Z., Rodriguez-Franco, M., Kraus, F., Bange, G. & Albers, S.-V. (2018). *Proc. Natl Acad. Sci. USA*, **115**, E1259–E1268. 10.1073/pnas.1716661115 PMC5819425 29358409

[bb58] Reindl, S., Ghosh, A., Williams, G. J., Lassak, K., Neiner, T., Henche, A. L., Albers, S.-V. & Tainer, J. A. (2013). *Mol. Cell*, **49**, 1069–1082. 10.1016/j.molcel.2013.01.014 PMC3615136 23416110

[bb59] Rudolph, J. & Oesterhelt, D. (1995). *EMBO J.* **14**, 667–673. 10.1002/j.1460-2075.1995.tb07045.x PMC398130 7882970

[bb60] Rudolph, J., Tolliday, N., Schmitt, C., Schuster, S. C. & Oesterhelt, D. (1995). *EMBO J.* **14**, 4249–4257. 10.1002/j.1460-2075.1995.tb00099.x PMC394508 7556066

[bb61] Sanna, M. G., Swanson, R. V., Bourret, R. B. & Simon, M. I. (1995). *Mol. Microbiol.* **15**, 1069–1079. 10.1111/j.1365-2958.1995.tb02282.x 7623663

[bb62] Schlesner, M., Miller, A., Besir, H., Aivaliotis, M., Streif, J., Scheffer, B., Siedler, F. & Oesterhelt, D. (2012). *BMC Microbiol.* **12**, 272. 10.1186/1471-2180-12-272 PMC3579733 23171228

[bb63] Schlesner, M., Miller, A., Streif, S., Staudinger, W. F., Müller, J., Scheffer, B., Siedler, F. & Oesterhelt, D. (2009). *BMC Microbiol.* **9**, 56. 10.1186/1471-2180-9-56 PMC2666748 19291314

[bb64] Silversmith, R. E. (2010). *Curr. Opin. Microbiol.* **13**, 177–183. 10.1016/j.mib.2010.01.004 PMC2862455 20133180

[bb65] Simms, S. A., Keane, M. G. & Stock, J. (1985). *J. Biol. Chem.* **260**, 10161–10168. 2991277

[bb66] Simms, S. A., Stock, A. M. & Stock, J. B. (1987). *J. Biol. Chem.* **262**, 8537–8543. 3298235

[bb67] Sircar, R., Greenswag, A. R., Bilwes, A. M., Gonzalez-Bonet, G. & Crane, B. R. (2013). *J. Biol. Chem.* **288**, 13493–13502. 10.1074/jbc.M112.445171 PMC3650386 23532838

[bb68] Speranskii, V. V., Metlina, A. L., Novikova, T. M. & Bakeyeva, L. Y. (1996). *Biophysics*, **41**, 167–173.

[bb69] Springer, W. R. & Koshland, D. E. Jr (1977). *Proc. Natl Acad. Sci. USA*, **74**, 533–537. 10.1073/pnas.74.2.533 PMC392324 322131

[bb70] Stock, J. B. & Koshland, D. E. Jr (1978). *Proc. Natl Acad. Sci. USA*, **75**, 3659–3663. 10.1073/pnas.75.8.3659 PMC392845 358191

[bb71] Streif, S., Staudinger, W. F., Marwan, W. & Oesterhelt, D. (2008). *J. Mol. Biol.* **384**, 1–8. 10.1016/j.jmb.2008.08.057 18786541

[bb72] Swanson, R. V., Sanna, M. G. & Simon, M. I. (1996). *J. Bacteriol.* **178**, 484–489. 10.1128/jb.178.2.484-489.1996 PMC177682 8550470

[bb73] Szilágyi, A. & Závodszky, P. (2000). *Structure*, **8**, 493–504. 10.1016/s0969-2126(00)00133-7 10801491

[bb74] Szurmant, H. & Ordal, G. W. (2004). *Microbiol. Mol. Biol. Rev.* **68**, 301–319. 10.1128/MMBR.68.2.301-319.2004 PMC419924 15187186

[bb75] Thomas, N. A. & Jarrell, K. F. (2001). *J. Bacteriol.* **183**, 7154–7164. 10.1128/JB.183.24.7154-7164.2001 PMC95564 11717274

[bb76] Thompson, J. D., Gibson, T. J., Plewniak, F., Jeanmougin, F. & Higgins, D. G. (1997). *Nucleic Acids Res.* **25**, 4876–4882. 10.1093/nar/25.24.4876 PMC147148 9396791

[bb77] Usher, K. C., De La Cruz, A. F., Dahlquist, F. W., Remington, S. J., Swanson, R. V. & Simon, M. I. (1998). *Protein Sci.* **7**, 403–412. 10.1002/pro.5560070221 PMC2143910 9521117

[bb78] Volz, K. & Matsumura, P. (1991). *J. Biol. Chem.* **266**, 15511–15519. 10.2210/pdb3chy/pdb 1869568

[bb79] Waterhouse, A. M., Procter, J. B., Martin, D. M., Clamp, M. & Barton, G. J. (2009). *Bioinformatics*, **25**, 1189–1191. 10.1093/bioinformatics/btp033 PMC2672624 19151095

[bb81] Welch, M., Oosawa, K., Aizawa, S. & Eisenbach, M. (1993). *Proc. Natl Acad. Sci. USA*, **90**, 8787–8791. 10.1073/pnas.90.19.8787 PMC47445 8415608

[bb82] Winter, G., Lobley, C. M. C. & Prince, S. M. (2013). *Acta Cryst.* D**69**, 1260–1273. 10.1107/S0907444913015308 PMC3689529 23793152

[bb83] Zhang, W., Brooun, A., McCandless, J., Banda, P. & Alam, M. (1996). *Proc. Natl Acad. Sci. USA*, **93**, 4649–4654. 10.1073/pnas.93.10.4649 PMC39333 8643458

[bb84] Zhu, X., Amsler, C. D., Volz, K. & Matsumura, P. (1996). *J. Bacteriol.* **178**, 4208–4215. 10.1128/jb.178.14.4208-4215.1996 PMC178179 8763950

[bb85] Zschiedrich, C. P., Keidel, V. & Szurmant, H. (2016). *J. Mol. Biol.* **428**, 3752–3775. 10.1016/j.jmb.2016.08.003 PMC5023499 27519796

