# Supplementary material for: Structure of the archaeal chemotaxis protein CheY in a domain-swapped dimeric conformation
Source: Acta Crystallogr F Struct Biol Commun. 2019 Aug 30;75(Pt 9):576–85. doi: 10.1107/S2053230X19010896 (PMC6718144; doi:10.1107/S2053230X19010896)
Supplement: Supplementary file 1 [file f-75-00576-sup1.pdf]

STRUCTURAL BIOLOGY
COMMUNICATIONS

Volume 75 (2019)

Supporting information for article:

Structure of the archaeal chemotaxis protein CheY in a domain-swapped dimeric conformation

Karthik Shivaji Paithankar, Mathias Enderle, David C Wirthensohn, Arthur Miller, Matthias Schlesner, Friedhelm Pfeiffer, Alexander Rittner, Martin Grininger and Dieter Oesterhelt

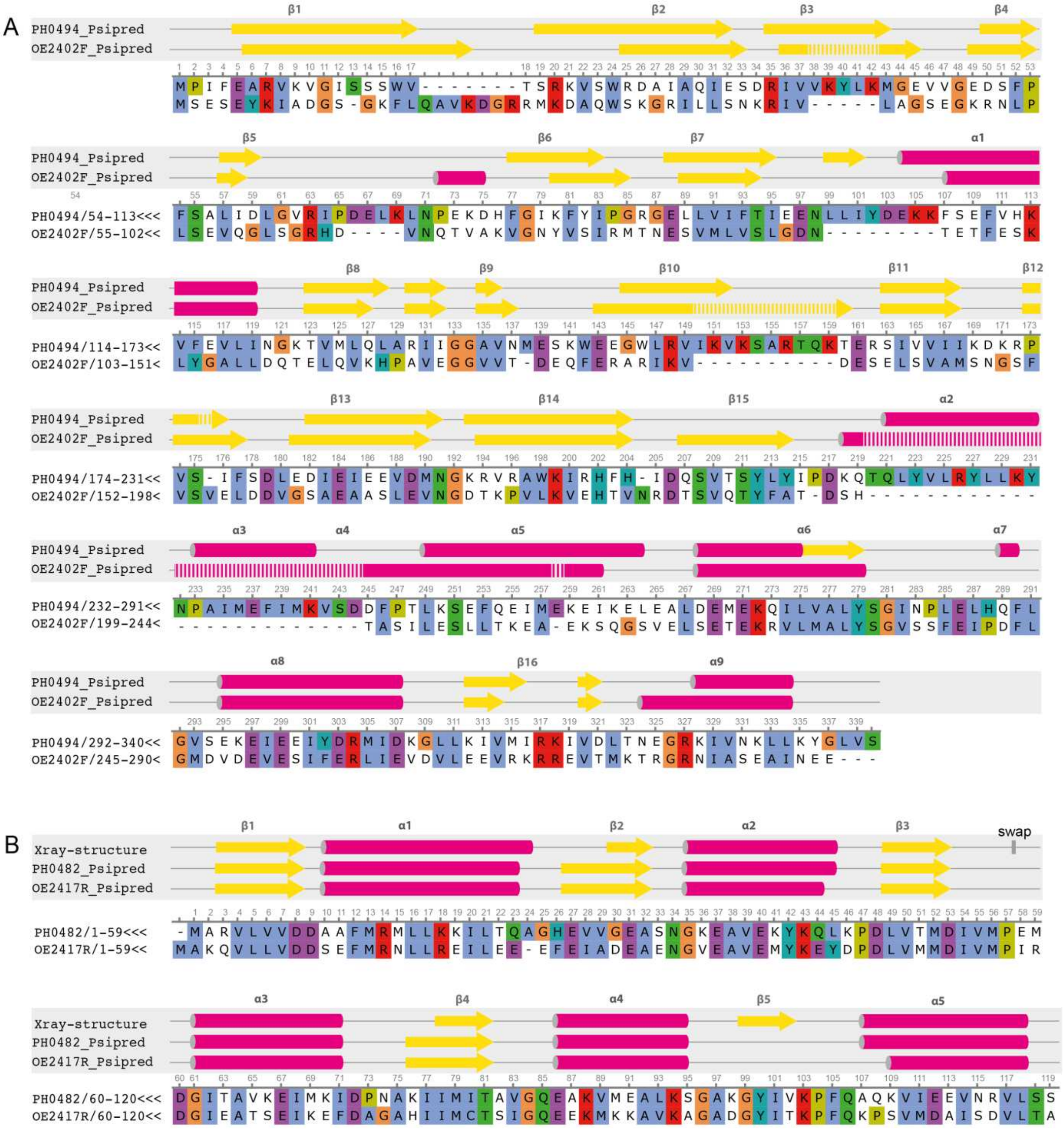

Fig. S1: Alignment of *P. horikoshii* and *H. salinarum* CheF and CheY. Alignment of *P. horikoshii* and *H. salinarum* CheF (A) and CheY (B). Sequence residue ranges are indicated. Sequences have been aligned with ClustalOmega (Thompson *et al.*, 1997) and are colored using ClustalX code. Helices are indicated by tube elements and lines show stretches with no secondary structure indicated/observed. Individual secondary structure predictions for the different sequences in the hub/spoke region were done using *PSIPRED* (Buchan *et al.*, 2013). For *P. horikoshii* CheY (B), the secondary structure pattern as observed in X-ray crystallographic data is indicated. The assignment of secondary structure was performed with *STRIDE* (Frishman & Argos, 1995). *H. salinarum* CheF shows deletions that are predicted to be located within loop regions of *P. horikoshii* CheF (residue ranges 99-106 and 150-159; numbering in *P. horikoshii* CheF equivalent positions), as well as to omit helices α3 and α4 (residue range 220-244; numbering in *P. horikoshii* CheF equivalent positions).

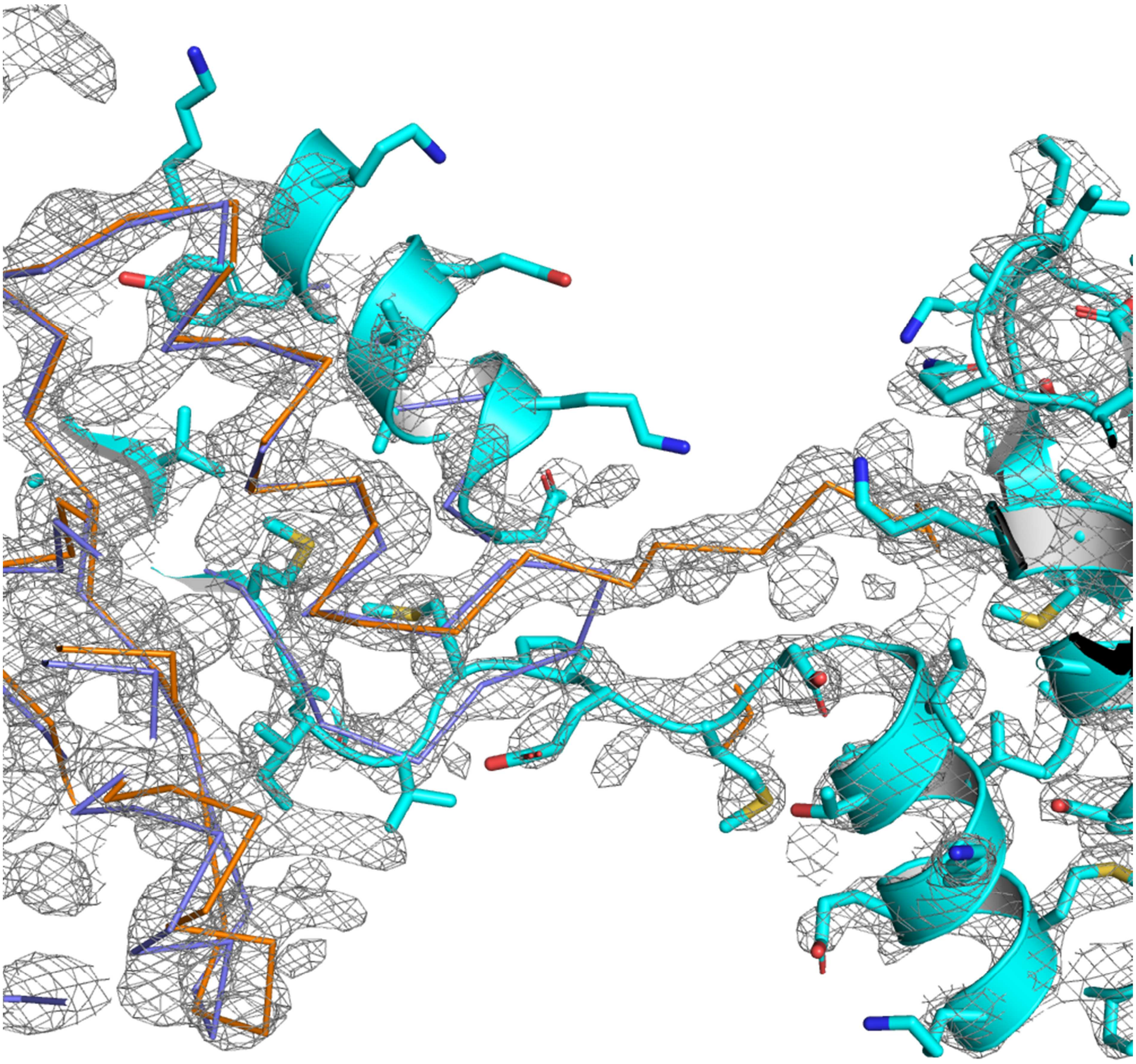

Figure S2: Electron density of the linker region. Iterative build composite omit maps at 2σ contour level are shown in grey. Chain A of PhCheY is coloured in cyan; and a symmetry equivalent of chain A is shown in gold. Shown in blue is pdb3tmy (CheY from *Thermotoga maritima*) superposed with its N-terminal (1-57) residues onto PhCheY (residues 1-57). Superposition performed with *lsqkab* within CCP4i; r.m.s.d of superposed residues 0.8 Å.

Supporting Note: Focus on Archaeal Flagellum

Cumulating evidence suggests that the archaeal motility system is structurally and evolutionarily different to the bacterial system and rather related to bacterial type IV pili (Faguy *et al.*, 1994, Cohen-Krausz & Trachtenberg, 2002, Jarrell & McBride, 2008); for example, the rotational movement of archaella and bacterial type IV pili are ATP-driven (Streif *et al.*, 2008, Kinosita *et al.*, 2016). The proteins constituting the archaellum have been termed archaellins (previously: flagellins). In the archaeal organism *Halobacterium salinarum*, archaellins are encoded by a multigene family (*arlA1+A2*, *arlB1+B2+B3* (previously *flgA1, flgA2, flgB1, flgB2* and *flgB3*) (Gerl & Sumper, 1988). The *arlB1-B3* genes cluster with genes *arlD,CE,FGHIJ* (previously *flaD,CE,FGHIJ*) which encode archaellar accessory proteins (Patenge *et al.*, 2001, Thomas & Jarrell, 2001). The exact role of several Arl proteins has been unraveled recently (reviewed in (Albers & Jarrell, 2015, 2018)). The ATPase ArlI and the transmembrane protein ArlJ belong to the same protein families as the pilus assembly ATPase PilB and the integral membrane protein PilC, respectively (Peabody *et al.*, 2003). There is clear evidence that ArlI is not only involved in archaella biogenesis, but is bifunctional, also being responsible for ATP-driven flagellar rotation (Ghosh *et al.*, 2011, Reindl *et al.*, 2013, Chaudhury *et al.*, 2016). ArlH (FlaH) is one of the conserved components of the archaeal motility system. Close homologs have not been detected in species that lack archaella, and the *arlH* gene is nearly always encoded adjacent to *arlI*. ArlH and ArlI interact with high affinity (Meshcheryakov & Wolf, 2016, Chaudhury *et al.*, 2018). The archaella accessory protein ArlF, which is homologous to ArlG, was implicated in stator function, as it was shown to interact with the S-layer in *Sulfolobus acidocaldarius* (Banerjee *et al.*, 2015). In Sulfolobus, the Crenarchaea specific accessory protein ArlX (FlaX) was shown to form a ring structure, which interacts with FlaH and FlaI (Banerjee *et al.*, 2013). In Euryarchaea, ArlX is replaced by the Euryarchaea-specific proteins ArlCDE, for which a more detailed characterization is still missing. The phylum-specificity of the euryarchaeal proteins may be correlated with two other Euryarchaea-specific traits: the existence of a polar cap and the usage of the Che system for chemotaxis (Speranskii *et al.*, 1996, Kupper *et al.*, 1994, Briegel *et al.*, 2017, Daum *et al.*, 2017).
